# Structural Health Monitoring (SHM) Study of Polymer Matrix Composite (PMC) Materials Using Nonlinear Vibration Methods Based on Embedded Piezoelectric Transducers

**DOI:** 10.3390/s23073677

**Published:** 2023-04-01

**Authors:** Loan Dolbachian, Walid Harizi, Zoheir Aboura

**Affiliations:** Centre de Recherche Royallieu, Roberval (Mechanics Energy and Electricity), Université de Technologie de Compiègne, CEDEX CS 60 319, 60 203 Compiègne, France; walid.harizi@utc.fr (W.H.); zoheir.aboura@utc.fr (Z.A.)

**Keywords:** polymer matrix composites (PMC), structural health monitoring (SHM), vibration analysis, nonlinear methods, in situ piezoelectric transducer

## Abstract

Nowadays, nonlinear vibration methods are increasingly used for the detection of damage mechanisms in polymer matrix composite (PMC) materials, which are anisotropic and heterogeneous. The originality of this study was the use of two nonlinear vibration methods to detect different types of damage within PMC through an in situ embedded polyvinylidene fluoride (PVDF) piezoelectric sensor. The two used methods are nonlinear resonance (NLR) and single frequency excitation (SFE). They were first tested on damage introduced during the manufacturing of the smart PMC plates, and second, on the damage that occurred after the manufacturing. The results show that both techniques are interesting, and probably a combination of them will be the best choice for SHM purposes. During the experimentation, an accelerometer was used, in order to validate the effectiveness of the integrated PVDF sensor.

## 1. Introduction

Nowadays, smart materials are an important field of research, and this kind of material should self-carry information about its health. The PMC materials are nice candidates for this purpose, as their use in industrial sectors is increasing due to their high mechanical properties [1,2,3,4,5,6,7,8,9,10]. In addition, the PMC can easily become smart through the embedment of sensors and actuators, such as piezoelectric transducers or optical fibers. These sensors can provide information about structural health monitoring (SHM) [11,12,13] and, also, about process monitoring (PM) [14,15,16], which makes these sensors a useful tool to detect defects during the manufacturing process.

PMC is a complex material, made of fiber and matrix, heterogeneous and anisotropic, so the damage mechanisms are complicated. The first type of damage that occurs in a composite material is transverse microcracks, which slowly propagate to the fibers according to the stress increase. Once the cracks reach the fibers, they follow the path of least resistance, which is the interface between fiber and matrix, thus creating the interfacial debonding phenomenon. This damage mechanism keeps propagating with the cracks and leads to a structure maintained only by the fibers. Increasing stress on the fibers breaks them, and the PMC structure fails with a combination of these damage mechanisms.

The dynamic elastic behavior can be influenced by these types of damage so that it cannot be explained by classical nonlinear models. The nonlinear elastic behavior can express itself in several ways during the dynamic wave propagation in a material. For example, it can generate sub- and super-harmonics (SFE and vibro-acoustic modulation (VAM) [17]), shift the natural frequency as a function of the driving amplitude (NLR), affect wave attenuation, or lead to long-term effects. A new theoretical description has been proposed in articles [18,19], which includes terms to describe classical nonlinearity, hysteresis, and discrete material memory. This approach relates material stresses not only to strains and their derivatives but also to the temporal derivative of strains, in order to reproduce phenomena such as hysteresis and material memory caused by damage. The nonlinear and hysteresis modulus is defined as a combination of linear and nonlinear terms, where the change in amplitude of deformation over the last period, as well as the measure of material hysteresis, is also considered. In this new theoretical description, the stress σ and the strain ε are related as follows:

(1) $$\sigma =K\left(\varepsilon ,\dot{\varepsilon }\right).\varepsilon \;\;\;\;$$

(2) $$K\left(\varepsilon ,\dot{\varepsilon }\right)=K_{0}\left(1+\beta \varepsilon +\delta \varepsilon^{2}+\alpha \left(\Delta \varepsilon +\varepsilon \left(t\right)sign\left(\dot{\varepsilon }\right)+\ldots \right)\right)\;$$

where *K*_0_ is the linear modulus, *β* and *δ* are the classical quadratic and cubic nonlinear parameters, *α* is the nonlinear hysteretic parameter, and sign($\dot{\varepsilon }$) = –1 or 1 according to the sign of $\dot{\varepsilon }$.

These complex damage mechanisms are the reason why robust SHM methods should be found to accurately predict damage. Nowadays, the tendency is to use multi-acquisition methods (ultrasonic testing, acoustic emission, electrical capacitance, etc.) and data fusion. This article is testing the efficiency of two nonlinear vibration methods: nonlinear resonance (NLR) and single-frequency excitation (SFE). Both methods have shown interesting results in the literature, which makes them powerful candidates to become one part of this multi-acquisition process. The current tendency is to focus on nonlinear methods [20,21,22,23,24,25,26,27,28,29,30,31], as it has been well established in the literature that linear vibration methods are not sufficient to detect small damage in composite structures [32], hence the choice of such methods in this study. This article first introduces the smart material used in the experimentation and the damage creation; second, it describes the experimentation setup; third, it explains the concept of the two nonlinear methods; and finally, it presents the results and discussion of the methods that have been used.

## 2. Materials

### 2.1. PVDF Transducers and PMC Material

The PMC plates are made from the following:

- Six plies of glass fibers 2/2 twill fabric (thickness 0.2 mm, from *Gazechim Composites*);
- An orthophthalic unsaturated polyester resin (pre-accelerated) Norester 822 for infusion (from *Nord Composites),* with a degassing pressure of –0.4 bar during 4 min and an injection pressure of –0.8 bar during the LRI;
- 1% wt. of methyl ethyl ketone peroxide (MEKP) ketanox B180 (from *C.O.I.M s.p.a.*).

**Figure 1:**
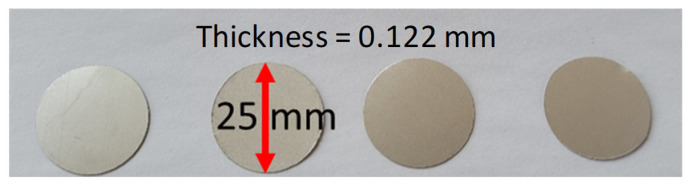
PVDF transducers cut into a disk shape.

In this study, polyvinylidene fluoride (PVDF, from TE Connectivity) transducers were used as sensors. The PVDF film thickness is 110 μm, and the total thickness is 122 μm due to the silver ink metallization. They were cut from an A4 sheet into a disk shape of 25 mm diameter (Figure 1). These transducers have lower piezoelectric properties than lead zirconate titanate (PZT) transducers, but they still are good candidates for embedment, as they have higher flexibility and can be placed in curvilinear shapes. However, its temperature range from –20 to +140 °C must be considered for SHM purposes.

These PVDF transducers were integrated into the middle plane of the PMC plates (between plies 3 and 4) using a liquid resin infusion (LRI) manufacturing method. The wiring was made from tinned copper wires of 210 μm diameter, and the static capacitance was tested for each of them before and after the manufacturing, to control if they were still usable. It was tested with an LCR bridge (HM8118 from Rohde & Schwarz) and the PVDF was considered usable if the static capacitance was similar before and after the LRI (around 500 pF for this diameter).

### 2.2. PVDF Transducers and PMC Material

**Figure 2:**
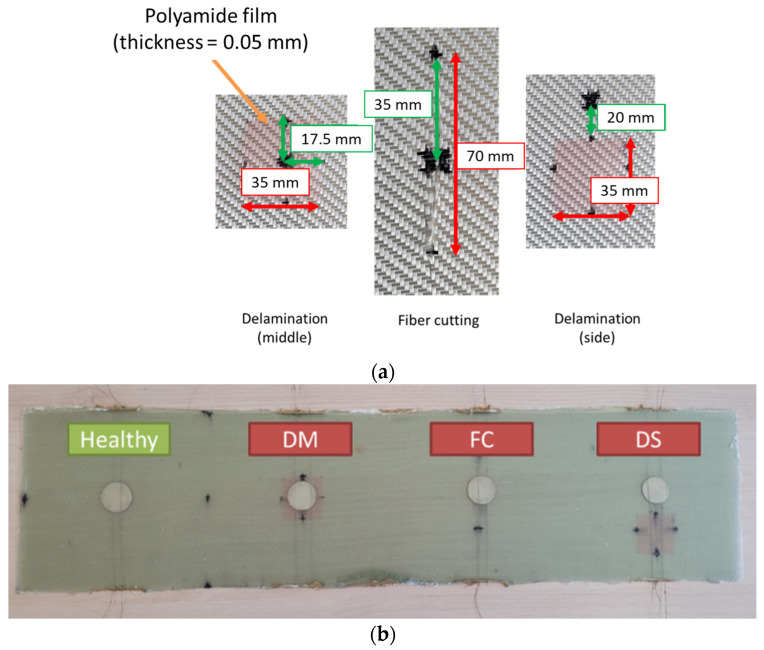
(**a**) Damage insertion and (**b**) manufactured plate before cutting.

In order to reduce the number of LRI operations, one big plate was manufactured with the dimensions of 620 × 150 × 1.5 mm^3^ and then cut into four 150 × 150 × 1.5 mm^3^ plates. One area of 150 × 150 mm² was used as a healthy area while three others with the same dimension were used to create damage. Two delaminations were introduced by inserting 35 × 35 × 0.05 mm^3^ polyamide film between plies 3 and 4 at two different positions: in the middle below the PVDF sensor (named DM) and on the side between the middle and the edge of the plate (named DS). One fiber cutting of 70 mm was made on the middle axe of the third ply. Each area has its integrated PVDF sensor in the middle plane (Figure 2).

**Figure 3:**
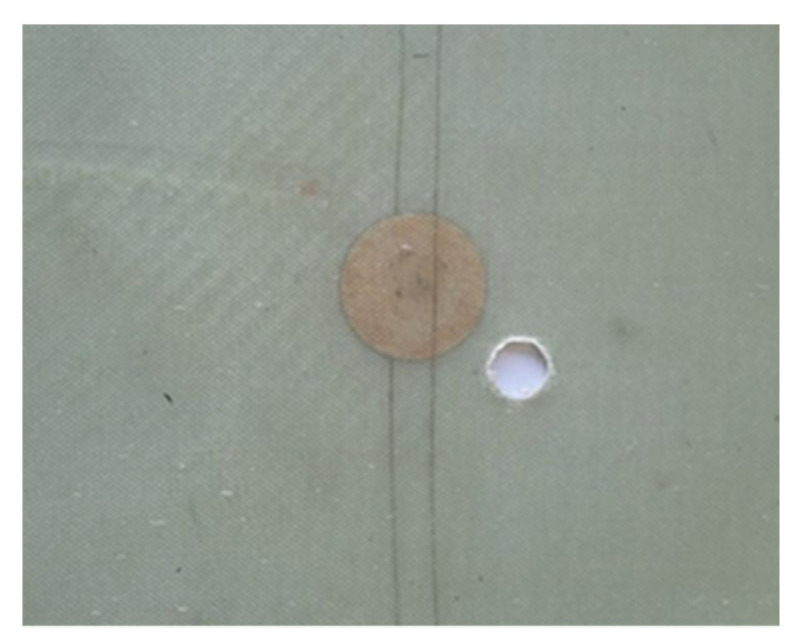
Smart PMC plate with a 10 mm diameter hole.

Once the LRI was finished and the cutting made, 4 smart PMC plates were usable for the vibration analysis. The vibration data were first obtained on the healthy plate. Later, a hole with an increasing diameter (1.5, 4, 6, 10 mm) was introduced with a series of vibration tests for each (Figure 3). The choice of the hole was made in order to easily compare with a numerical model. All the configurations are summarized in Table 1.

## 3. Experimental Vibration Setup

**Figure 4:**
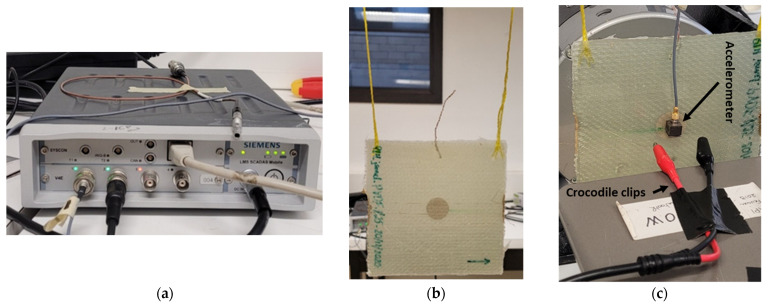
(**a**) Signal analyzer, (**b**) suspended plate, (**c**) crocodile clips and accelerometer.

**Figure 5:**
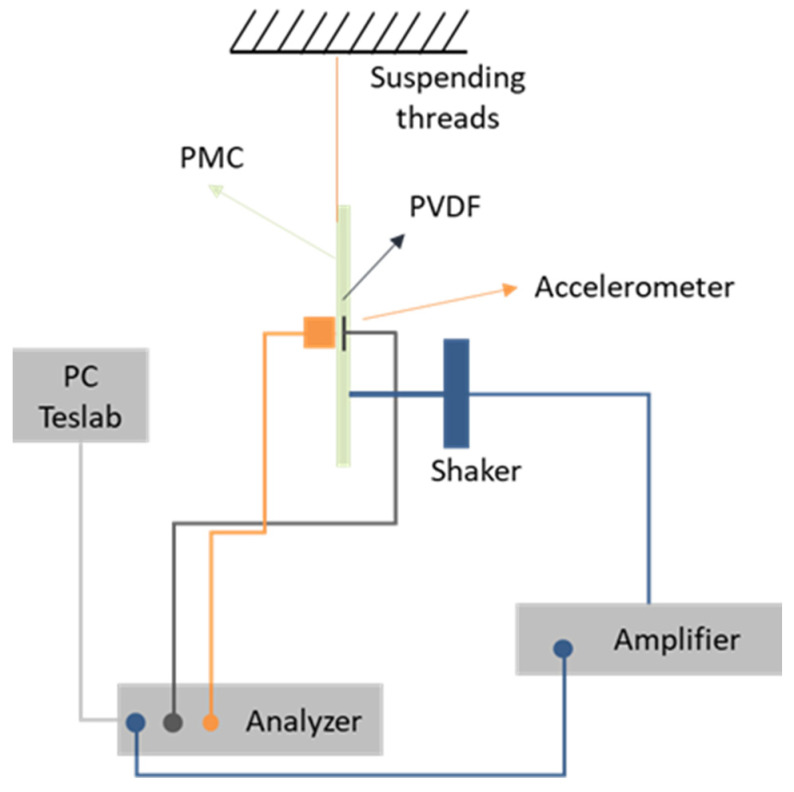
Schematic of the setup used for nonlinear vibration analysis.

To perform the vibration experimentation, the structure was suspended with two elastic threads in order to approximate the free-free boundary condition. The effectiveness of the PVDF as a vibration sensor has been tested and validated through several studies [33,34]. This work compared the results with those obtained by an accelerometer glued in the same position as the PVDF but on the surface. The output voltage of the PVDF was caught by two crocodile clips and connected to a signal analyzer (*Siemens*—LMS Scadas mobile 01). The equipment can be observed in Figure 4. This kind of analyzer directly manages the input and output data (fast Fourier transform (FFT) is automatically processed to visualize the result in the frequency domain) and allows observing the results in the Testlab software. It can also provide an electrical signal (signal generator). An electromechanical shaker (*Brüel & Kjaer*—Modal exciter type 4824) was used to excite the structure using a thin rod of 1mm diameter glued on the surface, in order to minimize surface excitation. In addition to this equipment, an amplifier (*Brüel & Kjaer*—Power Amplifier Type 2732) was used to provide the electrical signal delivered by the analyzer to the shaker. Figure 5 schematizes the experimental device used in the vibration tests.

**Figure 6:**
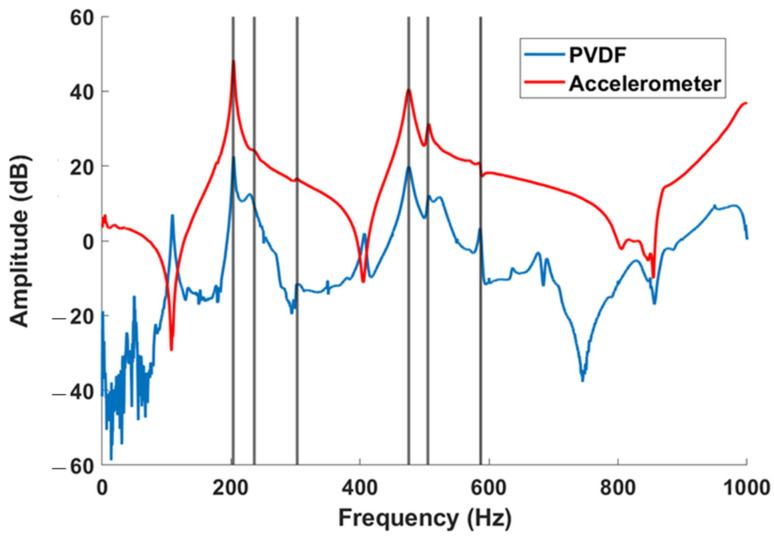
PVDF and accelerometer FRFs from impact hammer test; vertical lines represent some natural frequencies.

To validate the effectiveness of the PVDF, a hammer (*Brüel & Kjaer*—Impact hammer type 8206) was used to make the structure vibrate from a hit at a random location. The hammer has a cell force at its tip to store the input signal into the analyzer. By analyzing the frequency response function (FRF), the comparison between the two sensors showed a maximum deviation of tested natural frequencies (vertical lines) of 0.015% over the frequency range of 100–1000 Hz (Figure 6). The PVDF output was very noisy under 100 Hz. This test validated the use of the PVDF as a vibration sensor for the following experimentations. 

## 4. Nonlinear Methods

As composite structures are complex materials with complex damage mechanisms, the linear vibration methods are often insufficient to detect small damage. This is why the use of nonlinear methods has become more and more widespread in recent decades. Among these methods, two were chosen for this study: nonlinear resonance (NLR) and sub- and super-harmonics generation, or single frequency excitation (SFE).

### 4.1. Nonlinear Resonance

**Figure 7:**
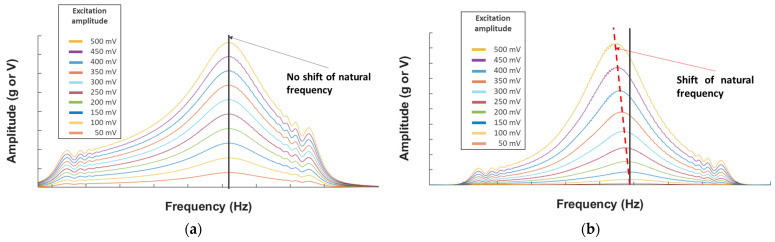
(**a**) Example of the NLR method for a healthy structure and (**b**) for a damaged structure.

In this technique, the structure is excited around its natural frequencies, with a sweep signal and increasing amplitude (for example from 100 mV to 1 V every 100 mV step). The natural frequency will be the same for each amplitude of excitation for an intact structure while a shift in frequency will be observed for a damaged structure (Figure 7). Several modes must be investigated in order to visualize which modes are influenced by the damage. This technique has been well studied and used in [20,28,29,35]. Some damage indicators and their combination can be used, such as the frequency shift (f_i_–f_0_) and the sequential frequency shift (f_i_–f_i−1_).

### 4.2. Sub- and Super-Harmonics Generation

**Figure 8:**
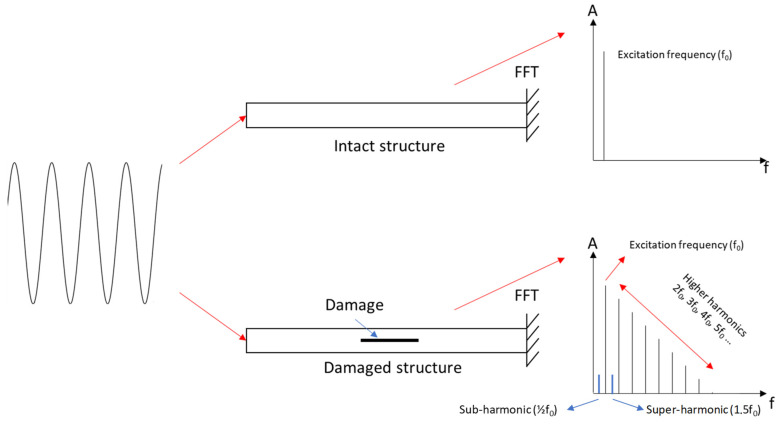
Sub- and super-harmonics generation method (adapted from [37]).

The sub- and super-harmonics generation method allows exciting the structure with a harmonic signal and observing only fundamental frequencies in the output of the healthy structure and a generation of sub- and super-harmonics in the damaged structure. Some researchers [36,37] used natural frequencies of the structure or a ratio of these natural frequencies (such as 1/3, 1/2, 2, etc.) as the frequency of excitation. In addition, two kinds of super-harmonics can be observed: the integer super-harmonics (2, 3, 4, etc.) and the super-harmonics built from a combination of the sub-harmonics and the integer super-harmonics (1.5, 2.5, 3.5, etc.) (Figure 8).

### 4.3. Experimental Protocol

Every experiment was repeated three times to check its repeatability, with a sampling frequency of 0.15 Hz. The protocol was as follows:

- Send a white noise at 0.5 V (out of amplifier) with a frequency range of 0–1200 Hz;
- Choose six natural frequencies from the FRF to investigate (this choice was made for the healthy plate regarding the results of PVDF and accelerometer in order to observe the best signals, and the same modes were used for the damaged plates);
  ○ The modes around 105, 160, 420, 540, 720, and 840 Hz;
  ○ These values are approximate, each plate and test has its own value (Table 2);
- Send a sweep signal with a frequency range of 40 Hz around the natural frequency excited and increase the amplitude of excitation 10 times (from 50 to 500 mV (out of amplifier));
- Send a harmonic signal corresponding to the frequency of the 1st, 2nd, and 4th modes of vibration (caught by the white noise) and their ratios 1/3, 1/2. The excitation level is 1 V (out of amplifier);
  ○ Around [105, 52.5, 35] Hz, [160, 80, 53.33] Hz and [420, 210, 140] Hz;
  ○ These values are approximate, each plate and test has its own value (Table 2);

The variation of natural frequencies from one plate to another is mainly due to differences in manufacturing (dimension, the proportion of resin/fiber, the position of the sensor, etc.), but also from the gluing of the rod and from the damage. This is why we choose not to use this comparison as a DI.

## 5. Results and Discussion

### 5.1. Nonlinear Resonance

**Figure 9:**
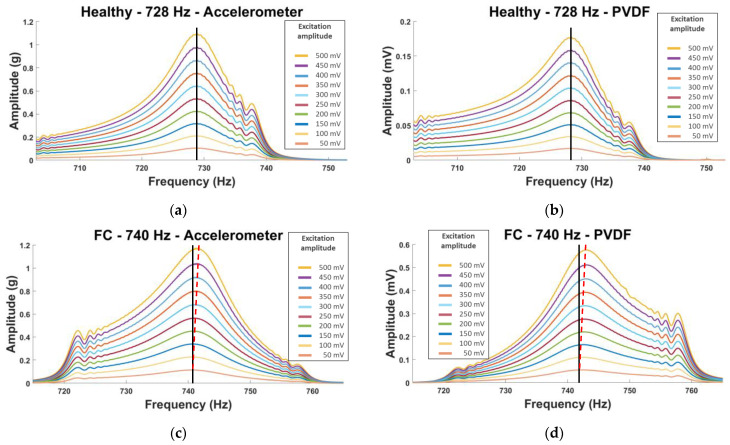
Amplitude vs. frequency curves for (**a**,**b**) healthy and (**c**,**d**) FC plates using the NLR method. (**a**,**c**) Accelerometer and (**b**,**d**) PVDF sensors.

To observe this phenomenon, we used the autopower linear [38] as the amplitude (an acceleration in ‘g’ unit (the gravitational acceleration on Earth = 9.80665 m.s^−2^) and a voltage according to the sensor used) versus frequency curves for each mode and plate. We can see in Figure 9 examples of healthy and FC plates. The lowest amplitude curve corresponds to the initial excitation, here 50 mV, and each increasing amplitude curve corresponds to an increment of 50 mV, up to 500 mV. 

On these curves, we can observe that the FC plate shows a small shift in frequency (red dotted line) for both sensors, while this is not the case for the healthy plate (black line). However, it is not a clear shift, and the visualization is not optimal. This is why the choice of the frequency shift parameter as a damage indicator (DI) has been made and visualized on histograms: frequency shift = (f_i_–f_0_). (f_i_ corresponds to the natural frequency of the i-*th* excitation and f_0_ to the initial one (here 50 mV)).

#### 5.1.1. Manufactured Damaged Plates

**Figure 10:**
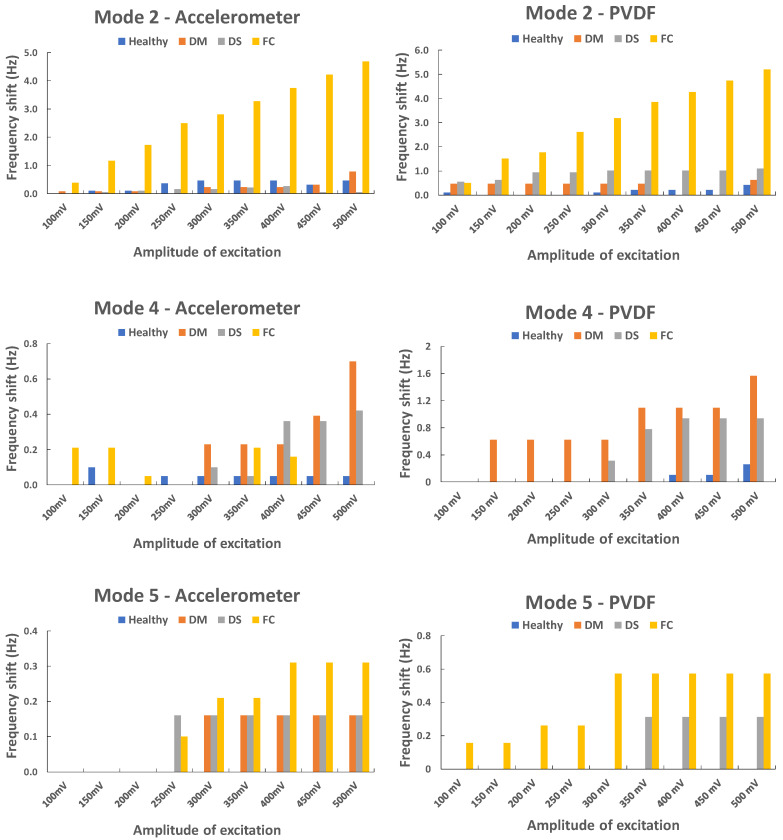
Frequency shift vs. amplitude of excitation: second, fourth, and fifth natural frequencies.

The DM, DS, and FC plates were compared with the healthy plate. However, the healthy plate for the first natural frequency showed a big shift, this is why this mode was not used for the comparison. Three modes (second, fourth, and fifth) showed interesting shifts (Figure 10).

**Figure 11:**
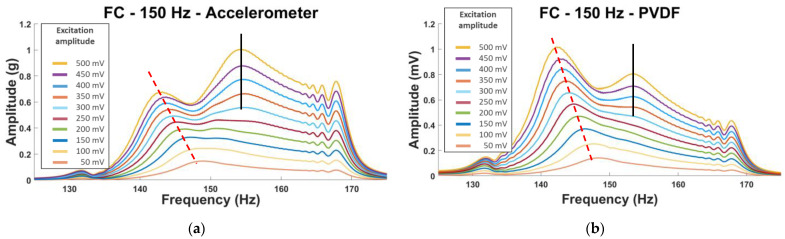
Second natural frequency of the FC plate using the NLR method. (**a**) Accelerometer and (**b**) PVDF sensor.

The fiber cutting (FC plate) seemed to influence the vibration of the second and the fifth modes while the two delaminations (DM and DS plates) seemed to influence the fourth mode, especially DM. The second mode was investigated for the FC plate, and an interesting phenomenon was observed. Two peaks are observable for this mode with one showing a big shift in frequency, a loss around 5 Hz (Figure 11); this phenomenon is observable for both sensors and every repeated test. The second peak is observable for the sixth level of excitation (300 mV) and higher, which means the plate does not show this nonlinear behavior at a low amplitude of excitation.

**Figure 12:**
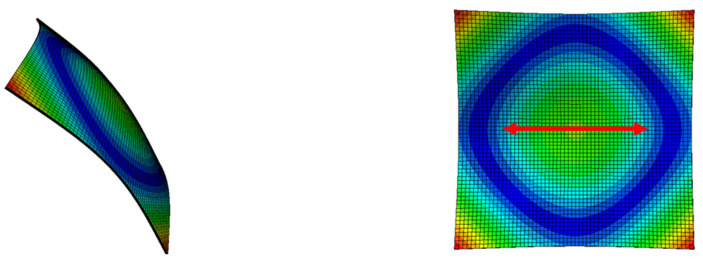
Second-mode shapes of a 150 × 150 × 1.5 mm^3^ plate from Abaqus software. (Red arrow = FC).

In the second mode, the vibration bends the plate area inside the blue “circle” in Figure 12, which corresponds to the fiber cutting location. This could create the nonlinearities in the vibration of the plate observed in Figure 11.

#### 5.1.2. Plate with Holes

**Figure 13:**
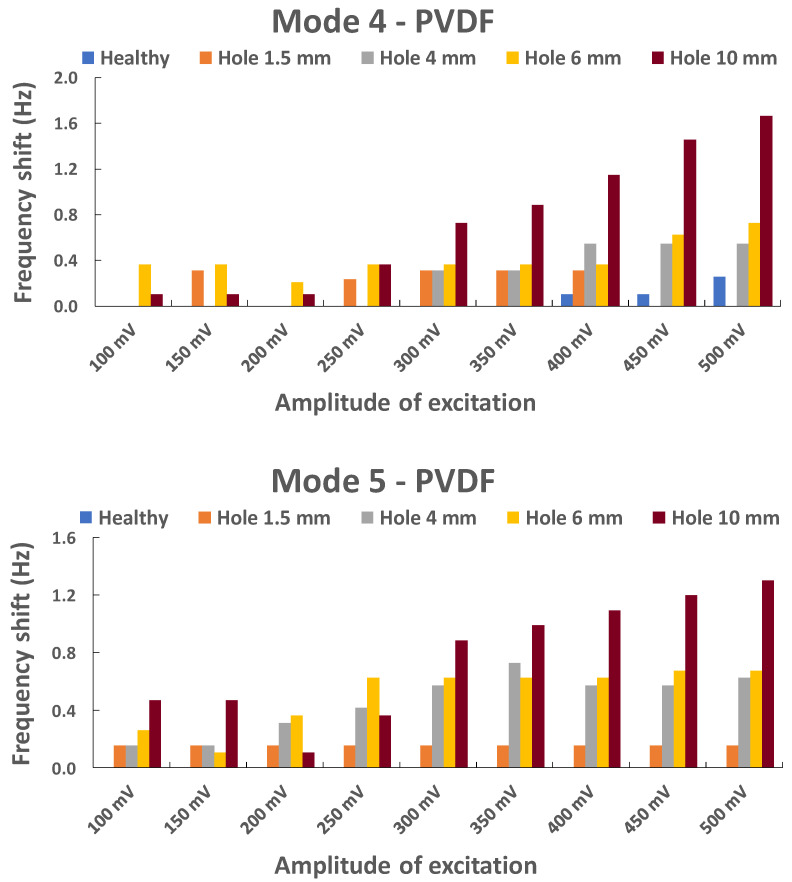
Evolution of the frequency shift in the fourth and fifth natural frequencies with an increasing hole diameter.

We recall that for this plate with incremental holes (1.5, 4, 6, and 10 mm), we will only study the response of the PVDF. Except for the first natural frequency, all the modes showed sensitivity to the presence of damage when it was greater than 4 mm in diameter. However, the fourth and fifth natural frequencies produced the best results (Figure 13). The vibration frequency offset increases with the increase of the excitation amplitude and the hole diameter, to reach significant values from 300 mV. It is clear that the 3 diameters of 4, 6, and 10mm generate more vibration frequency shifts than that of 1.5mm, especially in the 5th mode.

#### 5.1.3. Summary of the NLR Method

It is clear that this method is useful if the structure is vibrating around the influenced natural frequency (Table 3), which is different for every type of damage and localization. In service life, we consider that the damage is unknown, which means several modes must be tested. Every manufactured damage has shown its influence on one or two modes, which is encouraging regarding how small the damage is. The FC is the type of damage that shows the highest sensitivity for this method, which makes sense because in service life, the rupture of the fiber is the damage that is the most severe for the PMC structures.

### 5.2. Sub- and Super-Harmonics Generation

**Figure 14:**
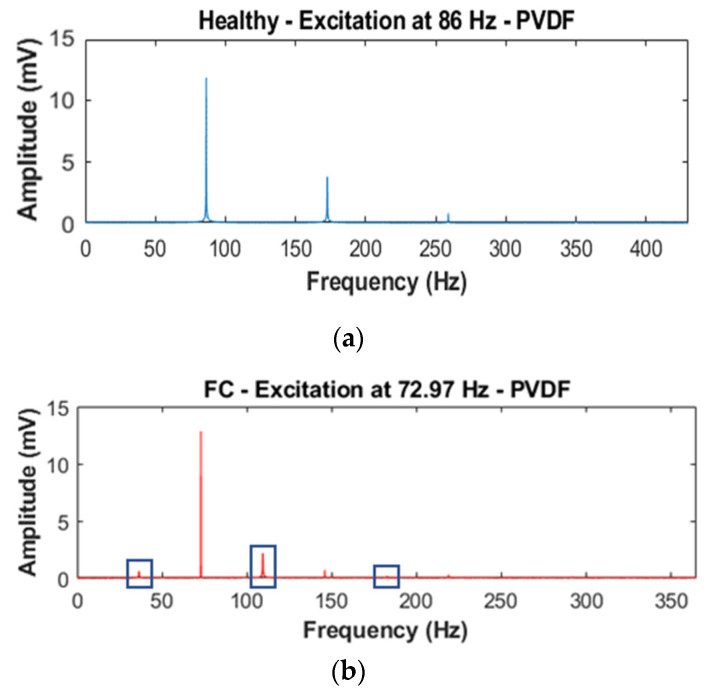
Amplitude vs. frequency curves for (**a**) healthy and (**b**) FC plates for the SFE method.

We observe the generation of sub- and super-harmonics on the amplitude of the output (g or V according to the sensor used) versus frequency curves for each mode and plate. Similarly, with the previous method, the visualization using this type of curve is not optimal: Figure 14 represents some peaks with very small amplitudes. This is why we propose to use the ratio R_peak_ (%) = 100 × A_peak_/A_fundamental_ as a DI where A_peak_: amplitude of the peak; A_fundamental_: amplitude of the fundamental. The first thing we noticed is that the healthy plate also showed integer super-harmonics (2f_fundamental_, 3f_fundamental, etc.)_, which means this cannot be used as a DI, but the sub-harmonics and their combination showed interesting results (on the FC plate curve in Figure 14, we can see these phenomena framed in blue). It is not uncommon to observe a difference between the accelerometer and PVDF when using this technique. As both do not catch the same output (voltage and acceleration), the amplitude will be different according to the frequency.

**Figure 15:**
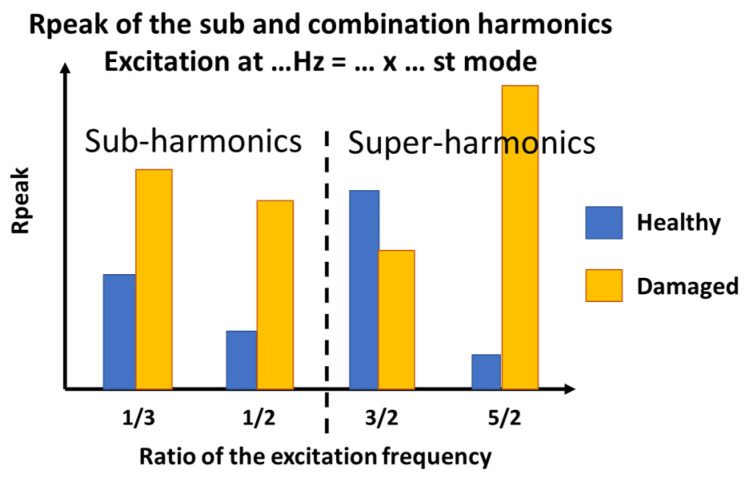
Histogram visualization and example for SFE method.

To visualize the influence of damage, a histogram showing the R_peak_ for each ratio of fundamental frequency excitation was created. The sub-harmonics 0.3/0.5 and the super-harmonics 1.5/2.5 were used. In order to visualize the ratio of the four plates, R_peak_ (%) is presented in a logarithmic scale (Figure 15).

In the example in Figure 15:

- The excitation frequency is 150 Hz = 1/2 × 3rd mode (the 3rd mode natural frequency is 300 Hz):
- The sub-harmonics (1/3 and 1/2): R_peak_ is calculated from the amplitude of the output peaks at 75 and 50 Hz;
- The super-harmonics (1.5 and 2.5): R_peak_ is calculated from the amplitude of the output peaks at 225 and 375 Hz;

#### 5.2.1. Manufactured Damaged Plates

**Figure 16:**
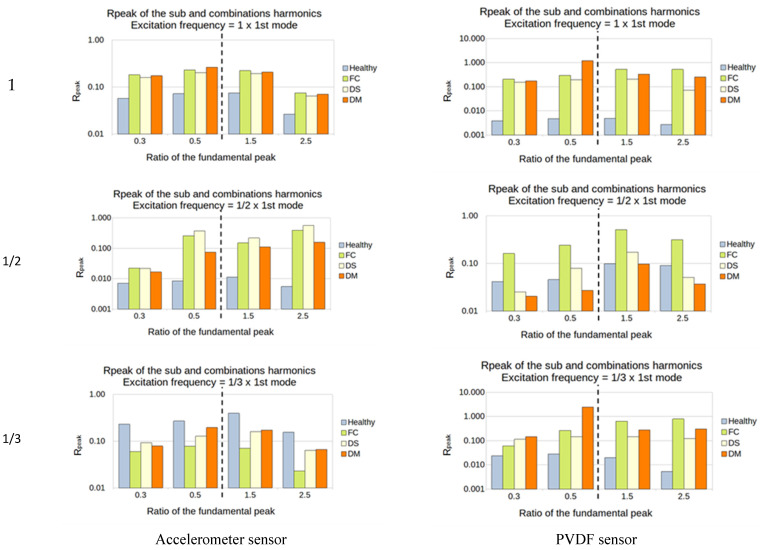
First natural frequency and its ratio of 1, 1/2, and 1/3 as the frequency of excitation.

Except for the accelerometer sensor for the excitation of 1/3, the ratio for all the other tests showed a high sensitivity to every type of damage and every sub- and super-harmonic observed, for the first natural frequency (Figure 16).

**Figure 17:**
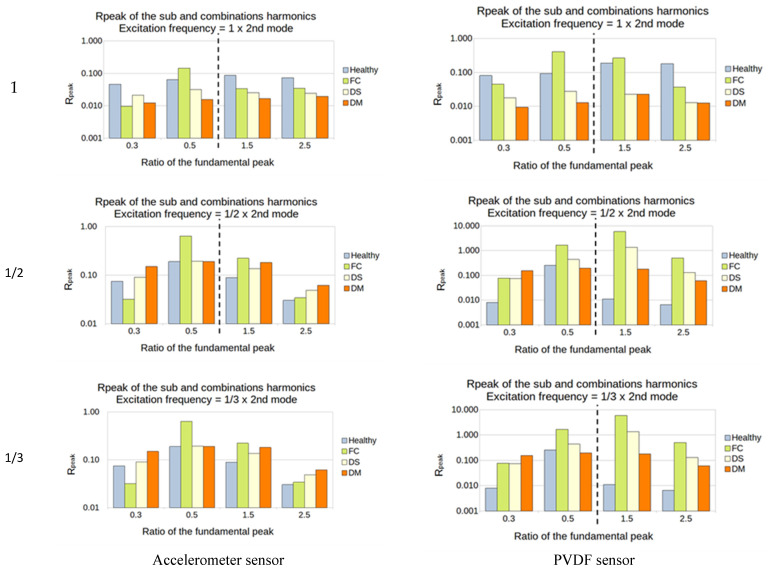
Second natural frequency and its ratio of 1, 1/2, and 1/3 as the frequency of excitation.

For the second natural frequency, the difference between healthy and damaged plates is smaller; indeed, only the FC plate showed a high sensitivity in almost all the cases. Another interesting thing is that the super-harmonics of 1.5 and 2.5 provide good results for the excitation frequencies of 1/2 and 1/3 for both sensors. No interesting results came out from the ratio of 1 (Figure 17). 

**Figure 18:**
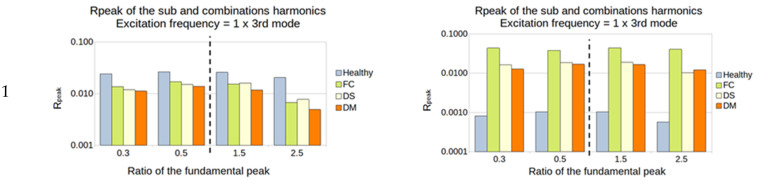

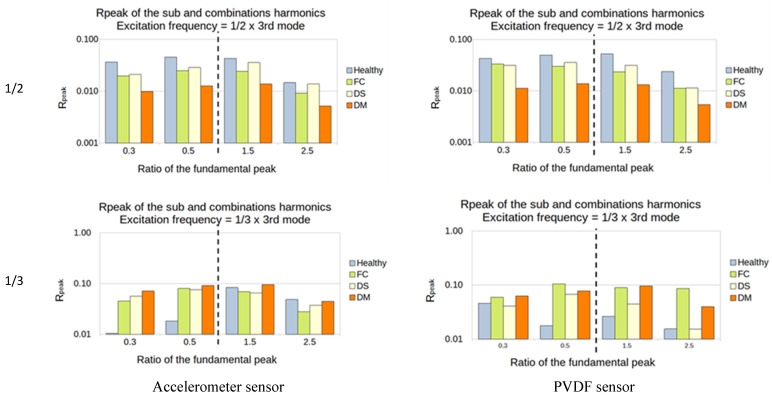
Third natural frequency and its ratio of 1, 1/2, and 1/3 as the frequency of excitation.

For the third natural frequency, the PVDF showed sensitivity in every sub- and super-harmonics generation for the 1 and 1/3 excitation, while the accelerometer showed sensitivity only for the 1/3. However, the value of the ratio was weak, between 0.01 and 0.1% (Figure 18).

The damage that accounted for higher sensitivity in this method is the FC. The first two natural frequencies seem to be the best choice to vibrate the structure, as they induced a higher output from the sensors. In most cases, the observation of the super-harmonics (1.5 and 2.5) produced a higher difference between the healthy and the damaged structure.

#### 5.2.2. Plates with a Hole

**Figure 19:**
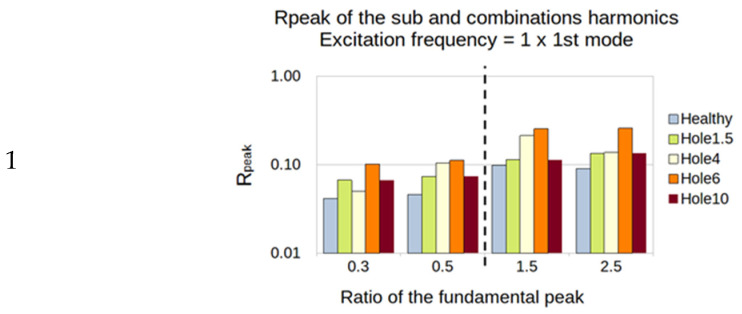

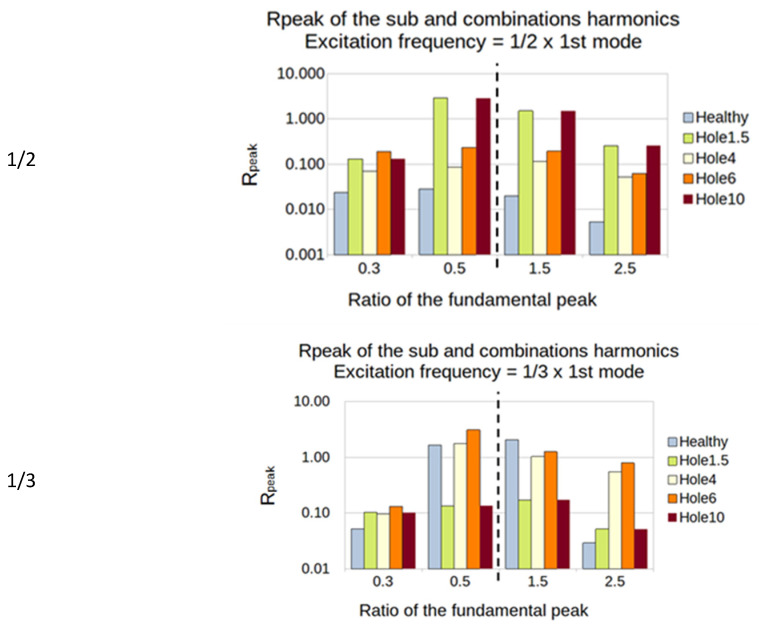
First natural frequency and its ratio of 1, 1/2, and 1/3 as the frequency of excitation for the PVDF sensor.

This is less clear for the hole than for the manufactured damage. However, for the first natural frequency, the ratio 1 of excitation showed sensitivity for every size of the hole and every sub- and super-harmonic. The ratio of 1/2 showed results, and the ratio of 1/3 did not show anything (Figure 19).

**Figure 20:**
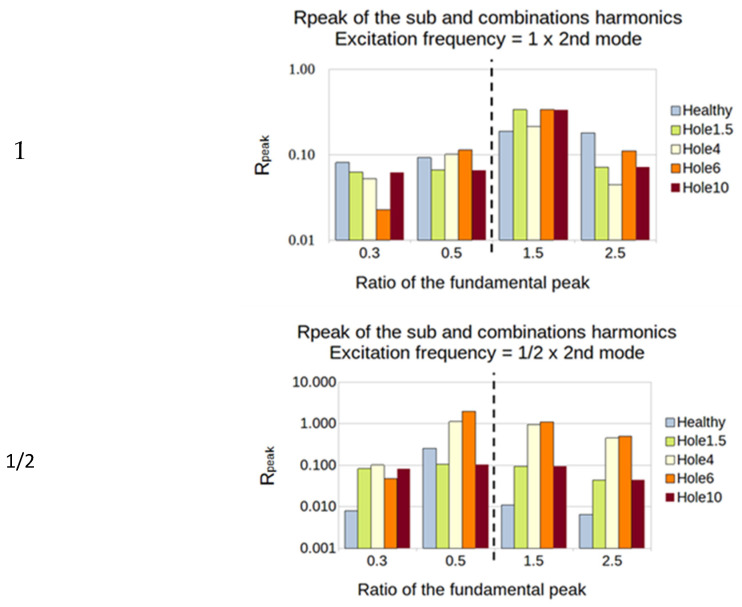

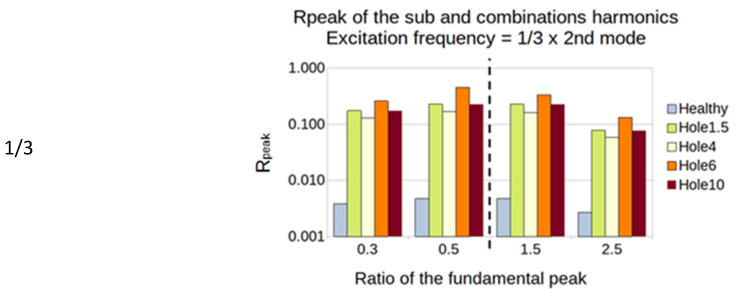
Second natural frequency and its ratio of 1, 1/2 and 1/3 as the frequency of excitation for the PVDF sensor.

For the second natural frequency, the PVDF showed a high sensitivity for ratio 1/2, especially on the super-harmonics. Both sensors showed a high sensitivity for the ratio of 1/3 for every size of the hole (Figure 20).

**Figure 21:**
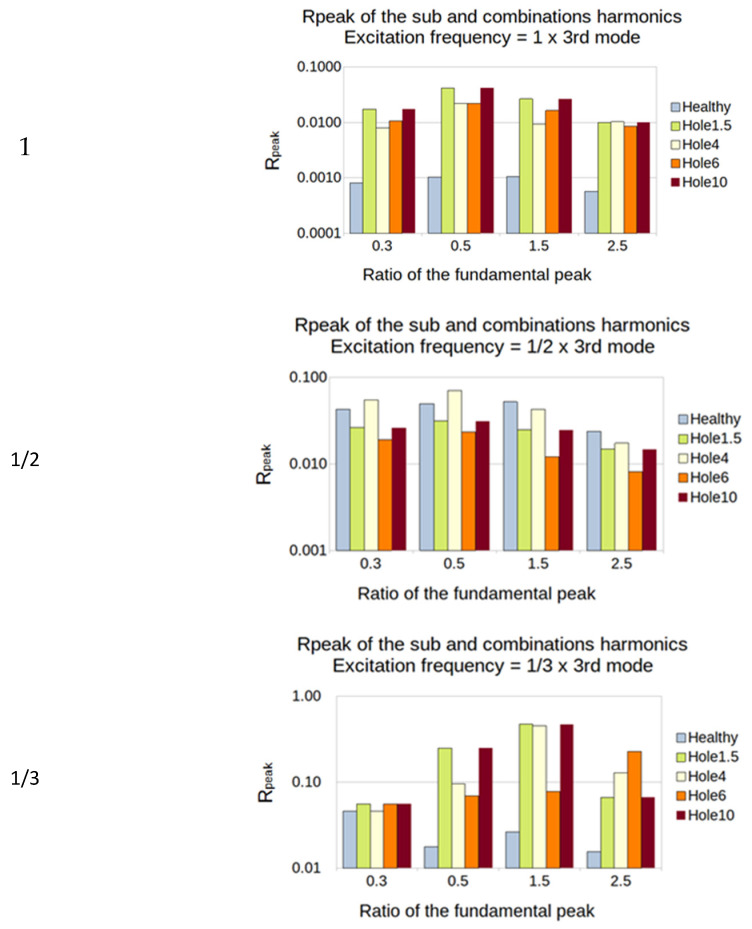
Third natural frequency and its ratio of 1, 1/2 and 1/3 as the frequency of excitation for the PVDF sensor.

For the third natural frequency, the PVDF showed a high sensitivity for the ratio of 1 and the ratio of 1/3, especially on the super-harmonics and the sub-harmonics 1/2 (Figure 21).

#### 5.2.3. Summary of the SFE Method

In Table 4 and Table 5, it is clear that the PVDF showed higher sensitivity than the accelerometer using this method. The manufactured damage seemed to influence the generation of sub-harmonics more than different holes.

## 6. Conclusions

In this article, both methods showed interesting results; however, the SFE method yielded the highest sensitivity and was easier to perform. The main advantage was that the information obtained for one test (2 sub-harmonics and 2 super-harmonics are used here) and the number of tests necessary to perform (9 tests in this experimentation) produced 36 interesting ratios for 9 tests to be compared. On the other hand, the NLR method offered the opportunity to check 54 ratios for 60 tests (which is time-consuming), and only the FC plate showed very high sensitivity. Since all of the manufactured damage was created in the middle plane of the plate, it could be interesting to investigate the effects of different types of damage at different thickness levels and at different locations.

Detecting damage in a composite structure is difficult, hence the choice of the nonlinear method. However, we observed that even healthy structures can exhibit nonlinear behavior, which is why special care should be taken when using these methods, especially to detect very small damage.

The variation of the frequency results between the accelerometer and the PVDF was small, and similar trends were observed with the NLR method for both sensors, which confirms the choice of such an in situ piezoelectric sensor for vibration analysis. In addition, the PVDF showed better sensitivity than the accelerometer for the SFE method, with higher amplitudes of sub- and super-harmonics. Another improvement could be making the structure fully autonomous; the actuation and the sensing could both come from piezoelectric transducers, and PZT seems more usable than the PVDF for actuation.

A nice future approach could be the combination of both methods or even VAM, to detect damage in a structure during its service life with a piezoelectric network. The transposition of this research from a laboratory scale to an operational scale will be a future challenge. Indeed, in service, structures have larger and more complex shapes, complex boundary conditions, environmental noises, etc. More modes could be tested in order to catch as much data as possible on a structure, and solutions should be found to avoid false alarms. Indeed, as the SFE method exhibits low amplitude peaks for the sub- and super-harmonics, external conditions could hide these phenomena or amplify them (for example if a noise overlaps with a sub- or super-harmonic). Automation of the NLR excitation with a higher amplitude could save a lot of time and make the method more efficient.

Nowadays, the tendency is to obtain information about damage through different kinds of testing, such as ultrasonic testing, acoustic emission, and electrical capacitance, and to use data fusion procedures to give a robust conclusion about the health of the structure. These nonlinear vibration methods used individually are not sufficient, but they can be part of a multi-acquisition process in the future.

## Figures and Tables

**Table 1:** The configuration of the 9 tested plates.

	Name	Description
**No damage**	Healthy	1 embedded PVDF;
**LRI** **damaged**	DM	1 embedded PVDF; 35 × 35 × 0.05 mm^3^ middle delamination between plies 3 and 4;
	DS	1 embedded PVDF; 35 × 35 × 0.05 mm^3^ side delamination between plies 3 and 4;
	FC	1 embedded PVDF; 70 mm middle fiber cutting of ply 3;
**Hole (mm)** **damaged**	1.5	1 embedded PVDF; 1.5 mm diameter hole drilling closed to PVDF;
	4	1 embedded PVDF; 4 mm diameter hole drilling closed to PVDF;
	6	1 embedded PVDF; 6 mm diameter hole drilling closed to PVDF;
	10	1 embedded PVDF; 10 mm diameter hole drilling closed to PVDF;

**Table 2:** Natural frequencies (Hz) of the 6 selected modes for the 8 plates.

Sensor	Natural Frequency	Healthy	DM	DS	FC	Hole (Diameter mm)			
						1.5	4	6	10
**Accelerometer**	**1**	107.7	108.5	115.8	107.8	106.6	106.1	104.9	105.4
	**2**	169.7	167.8	169.5	151.7	165.0	155.2	158.9	158.3
	**3**	414.4	413.2	423.9	423.4	417.7	417.6	415.6	414.2
	**4**	537.2	533.8	555.6	541.6	544.1	540.4	535.3	532.8
	**5**	730.2	720.6	760.8	732.5	730.9	731.9	728.9	727.4
	**6**	841.7	824.5	860.1	806.6	833.6	825.1	825.5	823.3
**PVDF**	**1**	108.4	111.5	118.6	111.5	109.6	108.4	107.4	107.3
	**2**	168.2	170.1	168.2	153.2	165.8	158.6	162.1	161.6
	**3**	414.2	417.2	426.8	428.4	420.4	419.2	416.6	415.6
	**4**	535.4	532.9	556.5	542.3	546.7	542.7	537.5	534.0
	**5**	729.3	719.6	766.5	739.2	734.8	732.3	730.7	729.6
	**6**	839.0	825.3	867.0	806.7	837.2	827.1	826.9	825.8

**Table 3:** Summary (X: not sensitive; O: sensitive; OO: very sensitive).

Damage	Natural Frequency					
	1st	2nd	3rd	4th	5th	6th
**FC**	X	OO	X	X	O	X
**DS**	X	X	X	O	X	X
**DM**	X	X	X	O	X	O
**Hole**	X	O	O	OO	OO	O

**Table 4:** Summary for PVDF (X: not sensitive (0 or 1 sub- or super-harmonic); O: sensitive (2 sub- or super-harmonics); OO: very sensitive (>2 sub- or super-harmonics)).

Damage	1st Frequency			2nd Frequency			3rd Frequency		
	1/3	1/2	1	1/3	1/2	1	1/3	1/2	1
**FC**	OO	OO	OO	OO	OO	O	OO	X	OO
**DS**	OO	O	OO	OO	OO	X	O	X	OO
**DM**	OO	X	OO	OO	OO	X	OO	X	OO
**Hole**	X	OO	OO	OO	OO	X	OO	X	OO

**Table 5:** Summary for accelerometer (X: not sensitive (0 or 1 sub- or super-harmonic); O: sensitive (2 sub- or super-harmonics); OO: very sensitive (>2 sub- or super-harmonics)).

Damage	1st Frequency			2nd Frequency			3rd Frequency		
	1/3	1/2	1	1/3	1/2	1	1/3	1/2	1
**FC**	X	O	O	O	O	X	O	X	X
**DS**	X	O	O	O	O	X	O	X	X
**DM**	X	O	O	O	O	X	O	X	X

## Data Availability

The data required to reproduce these findings cannot be shared at thistime as the data also form part of an ongoing study.
